# The Diseases of Historical Characters

**Published:** 1908-03-07

**Authors:** 


### The Diseases of Historical Characters.

Biography is a branch of literature that should specially commend itself to medical men. Our profession, like that of the lawyer, deals primarily with men, though sometimes we run a risk of forgetting this truth and assume that it concerns itself firstly and almost exclusively with disease, and its protean manifestations. But a knowledge of humanity, the true knowledge of man, is imperatively necessary for him who desires to become a good doctor, or even a great one. And to a large extent biography, though it can never replace personal observation and experience, will teach the searcher after truth many valuable lessons, not only about men and women, but also about their ailments and the effects of these. It is truly astonishing that no one has as yet attempted to devote himself to the elucidation of the diseases of great men. The subject, indeed, is an interesting one, and opens up illimitable vistas of pleasurable anticipation. What theories might not be built upon the symptomatology which has come down to us, the "selbst-diagnose" of one or other of our heroes! What mysteries remain to be discussed, unfolded, and unravelled by the enthusiastic searcher! The Duchess of Orleans, according to Dr. Gee, died of perforated gastric ulcer; Napoleon of carcinoma ventriculi. But there are many others whose ailments demand and deserve a somewhat closer examination than has been given them hitherto. Even in cases where the history is more or less clearly given there is room for further comment and elucidation. Milton, for instance, in the famous Latin epistle to Deodati, has described his initial symptoms in a masterly and vivid fashion, yet science has not yet proved whether

The drop serene that quenched my sight was a glaucoma or a cataract. Montaigne, in one of his essays, has given an equally clear and lucid description of his attacks of colic, yet it might be worth while to discuss whether he suffered from biliary or renal lithiasis. Besides these, a vast number of historical characters await an explanation of their diseases, and the obscurities of their clinical histories should afford interesting material for a medico-psychological study. What, for instance, did Schumann, the musician, die of? What Mozart, whose tragic death is a mystery which has lately exercised the Paris Academy of Medicine? What Charles II. (whose dying draught, compounded from a prescription of twenty Court physicians, is an awful example of empirical polypharmacy which throws an interesting sidelight upon the medical treatment of Royalty in his day), what Pope Honorius, what the Venerable Bede, Addison, Cromwell, Spinoza, and a host of other historical characters whose last moments are shrouded with a veil that no medical commentator has as yet presumed to lift?

It may be alleged that such an inquiry into the mysterious exits of our great men would be productive of no practical utility. What's Hecuba to us? But a conception of the real difficulties under which a life's work was accomplished adds greatly to the admiration or otherwise with which the student has invested his characters in imagination. Moreover, a clear understanding, where such is possible, of these cases will in many instances furnish a clue to biographical matters which seem difficult or inexplicable. The psychological study of Wagner, for example, is one of the most interesting that presents itself to the student of this great artist, and there is no doubt that an equally interesting study awaits the sympathetic critic who investigates the life and work of Schumann from the point of view of the alienist. That such study may be carried to excess is, of course, a danger that cannot be disregarded. There is a tendency, particularly noticeable in the apologists for one or other of the great geniuses that are separated by a very thin line from the class of degenerates, to go to unnecessary lengths in the psychological study of their admirations. The biographer who attempts such a task should be an expert in mental medicine, otherwise his attempt is a failure and his apology a fraud upon a public that bans without discrimination and forgives without understanding. Nevertheless the clinical study of the diseases of historical characters holds out many attractions that should make it sufficiently interesting for a medical commentator to try his hand at an attempt at elucidation.

